# 4015 Evaluation of the Impact of a Clinical and Translational Science Predoctoral Program on Post-Graduate Outcomes

**DOI:** 10.1017/cts.2020.212

**Published:** 2020-07-29

**Authors:** Alexandra Joelle Greenberg-Worisek, Katherine Cornelius, Becca Gas, Carmen Silvano, Karen Marie Weavers, Lewis R Roberts, Stephen C Ekker, Felicity Enders, Anthony Windebank

**Affiliations:** 1 Mayo Clinic

## Abstract

OBJECTIVES/GOALS: The Mayo Clinic Clinical and Translational Science (CTS) Predoctoral program aims to develop independent researchers capable of leading multi-disciplinary teams to accelerate the translation of discovery to application. Here, we detail the outcomes of our graduates over the past ten years (2010-2019). METHODS/STUDY POPULATION:): A survey was fielded with all CTS graduates whose degrees were conferred since the program’s inception to 2019. Items addressed their current position, whether they were still involved in research, what type of research they were involved in, and whether they stayed involved with education. They also submitted a recent CV, from which data were collected about publications and grants. A subset were then contacted for a semi-structured interview. Items included questions addressing motivation for pursuing a PhD in CTS, whether the program prepared them for their current work, gaps they felt they had in training, and whether they felt they were making a difference in the lives of patients. RESULTS/ANTICIPATED RESULTS: Of the 41 alumni, 34 responded (83% response rate). Of these, 19 (56%) are at Mayo Clinic, 9 (26%) work for other academic institutions, and 6 (21%) do not work for an academic institution. Most have remained in research (33/34, 97%). The majority (22/33, 67%) are involved in clinical research, 30% (10/33) in basic science, and 24% (8/33) in healthcare delivery research. Most (23/34, 68%) are engaged in educational activities. When asked about changes they have led, 67% (18/27) led quality improvement projects and 44% (12/27) designed a new research method. Several hold leadership positions either in their organization (12/16, 75%) or in a professional organization (10/16, 63%). DISCUSSION/SIGNIFICANCE OF IMPACT: The CTS Predoctoral program successfully prepares scholars for careers involving clinical and translational research; furthermore, alumni remain in research-oriented careers after graduation. We will continue to gather longitudinal data alumni move forward in their careers.

